# Profiling the molecular and clinical landscape of glioblastoma utilizing the Oncology Research Information Exchange Network brain cancer database

**DOI:** 10.1093/noajnl/vdae046

**Published:** 2024-03-27

**Authors:** Alexandra N Demetriou, Frances Chow, David W Craig, Michelle G Webb, D Ryan Ormond, James Battiste, Arnab Chakravarti, Howard Colman, John L Villano, Bryan P Schneider, James K C Liu, Michelle L Churchman, Gabriel Zada

**Affiliations:** Keck School of Medicine, University of Southern California (USC), Los Angeles, California, USA; Norris Comprehensive Cancer Center, University of Southern California, Los Angeles, California, USA; Department of Integrative Translational Sciences, City of Hope, Duarte, California, USA; Department of Integrative Translational Sciences, City of Hope, Duarte, California, USA; Department of Neurosurgery, University of Colorado School of Medicine, Aurora, Colorado, USA; Stephenson Cancer Center, University of Oklahoma Health Sciences Center, Oklahoma City, Oklahoma, USA; Department of Radiation Oncology, College of Medicine at The Ohio State University, Columbus, Ohio, USA; Huntsman Cancer Institute and Department of Neurosurgery, University of Utah, Salt Lake City, Utah, USA; Department of Internal Medicine, University of Kentucky College of Medicine, Lexington, Kentucky, USA; Department of Hematology/Oncology, Indiana University School of Medicine, Indianapolis, Indiana, USA; Department of Neuro-Oncology, Moffitt Cancer Center, Tampa, Florida, USA; Aster Insights, Hudson, Florida, USA; Department of Neurological Surgery, Keck School of Medicine of USC, Los Angeles, California, USA

**Keywords:** EGFR, glioblastoma, genomics, survival, transcriptomics

## Abstract

**Background:**

Glioblastoma exhibits aggressive growth and poor outcomes despite treatment, and its marked variability renders therapeutic design and prognostication challenging. The Oncology Research Information Exchange Network (ORIEN) database contains complementary clinical, genomic, and transcriptomic profiling of 206 glioblastoma patients, providing opportunities to identify novel associations between molecular features and clinical outcomes.

**Methods:**

Survival analyses were performed using the Logrank test, and clinical features were evaluated using Wilcoxon and chi-squared tests with *q*-values derived via Benjamini-Hochberg correction. Mutational analyses utilized sample-level enrichments from whole exome sequencing data, and statistical tests were performed using the one-sided Fisher Exact test with Benjamini-Hochberg correction. Transcriptomic analyses utilized a student’s *t*-test with Benjamini-Hochberg correction. Expression fold changes were processed with Ingenuity Pathway Analysis to determine pathway-level alterations between groups.

**Results:**

Key findings include an association of *MUC17, SYNE1,* and *TENM1* mutations with prolonged overall survival (OS); decreased OS associated with higher *epithelial growth factor receptor (EGFR)* mRNA expression, but not with *EGFR* amplification or mutation; a 14-transcript signature associated with OS > 2 years; and 2 transcripts associated with OS < 1 year.

**Conclusions:**

Herein, we report the first clinical, genomic, and transcriptomic analysis of ORIEN glioblastoma cases, incorporating sample reclassification under updated 2021 diagnostic criteria. These findings create multiple avenues for further investigation and reinforce the value of multi-institutional consortia such as ORIEN in deepening our knowledge of intractable diseases such as glioblastoma.

**Key Points**

- *MUC17, SYNE1,* and *TENM1* mutations are associated with longer overall survival (OS).
- Patients with OS < 1 year demonstrate increased expression of *TMBIM1* and *CLSTN1.*
- Fourteen transcripts are associated with OS > 2 years.

**Importance of the Study**

This study represents the first analysis of 206 glioblastoma cases documented in the Oncology Research Information Exchange Network brain cancer database. All cases were reclassified under new 2021 World Health Organization diagnostic criteria prior to analysis to evaluate hallmarks of glioblastoma through an updated lens and identify novel trends when histologically diagnosed cases are viewed in combination with molecularly diagnosed glioblastoma. This study identifies 3 genes (*MUC17, SYNE1,* and *TENM1*) in which mutations are associated with prolonged overall survival (OS), 14 transcripts (*NAJB5, PHTF2, TIPRL, CDC23, PGRMC2, CDKN2A, EXOSC9, MIS18BP1, RFC4, CNOT6, IQGAP2, AP3M1, ZNF521,* and *EPC1*) associated with OS > 2 years, and two transcripts (*TMBIM1* and *CLSTN1*) associated with OS < 1 year. Furthermore, the analysis characterizes the landscape of *epithelial growth factor receptor* alterations commonly seen in glioblastoma and suggests that increased EGFR mRNA expression, rather than amplification or mutation, correlates more strongly with OS.

Glioblastoma is the most common primary malignant brain tumor in adults.^1^ Despite decades of research efforts, median overall survival remains 12–18 months and less than 5% of patients survive five years.^2^ To address existing gaps in therapeutic design and clinical management, modern research efforts have increasingly evolved toward interrogating molecular mechanisms underpinning glioblastoma’s clinical course and inter-patient variability.

Some of the most well-established molecular features of glioblastoma include mutations in the telomerase reverse transcriptase *(TERT)* promoter region, phosphatase and tensin homolog *(PTEN),* and tumor protein 53 (*TP53),* alterations to the epithelial growth factor receptor *(EGFR*) gene, and chromosome-level changes such as combined gain of chromosome 7 and loss of chromosome 10.^3^ According to the 2021 World Health Organization Classification of Tumors of the Central Nervous System (WHO CNS) guidelines, diagnosis of glioblastoma is reserved for isocitrate dehydrogenase (*IDH)*-wild-type cases and can be made via histological characteristics including microvascular proliferation and necrosis surrounded by pseudopalisading cells.^4^ Alternatively, patients with histologically low-grade, *IDH*-wild-type astrocytoma may be diagnosed with glioblastoma if their tumor harbors a *TERT* promoter mutation, *EGFR* amplification, or combined gain of chromosome 7 and loss of chromosome 10.^4^ Shifts in diagnostic paradigms toward utilizing molecular features to complement or supersede histological interpretation underscore the integral role of molecular analysis in glioblastoma management.

The Oncology Research Information Exchange Network (ORIEN) database was established by a cohort of NCI-recognized comprehensive cancer centers for the purpose of advancing cancer research by leveraging molecular, clinical, and demographic data derived from hundreds of thousands of patients across multiple North American institutions. Herein we report the first analysis of this glioblastoma dataset, which integrates clinical, genomic, and transcriptomic analyses and incorporates case reclassification under 2021 WHO CNS guidelines to provide an updated, comprehensive understanding of the landscape of glioblastoma.

## Materials and Methods

### Patient Enrollment

Patients were enrolled in a Total Cancer Care® protocol (NCT03977402) implemented at the 18 cancer centers participating in the ORIEN consortium (see Author Note). Each patient was enrolled using an IRB-approved written informed consent at their treating institution. Tumor and germline samples from each patient were aggregated, sequenced, and harmonized by Aster Insights (Hudson, Florida, USA; Supplementary Material).

### Case selection and Reclassification

**Figure 1.**
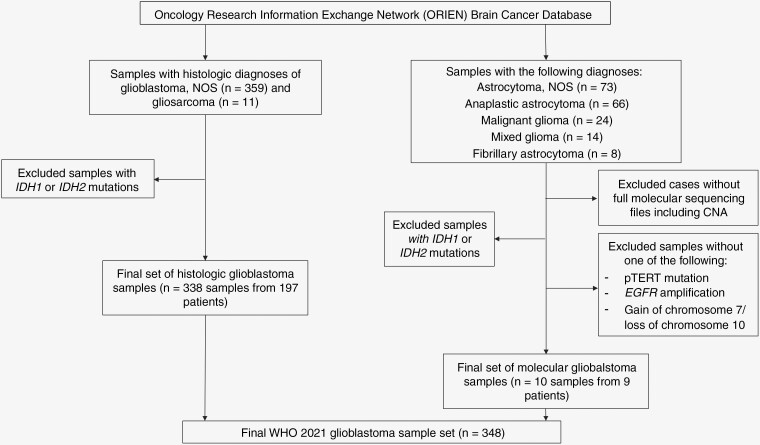
Glioblastoma case reclassification. Consort diagram depicting inclusion and exclusion criteria for this analysis. (NOS, not otherwise specified; IDH, isocitrate dehydrogenase; CNA, copy number alteration; pTERT, telomerase reverse transcriptase promoter; EGFR, epidermal growth factor receptor).

Glioma cases were selected from the ORIEN brain cancer database and grouped by diagnosis. Given that most diagnoses were established prior to 2021, cases were reclassified according to 2021 WHO CNS diagnostic criteria to select for glioblastoma based on histologic and/or molecular parameters (Figure 1).^4^ Information on molecular reclassification is available in Supplementary Material.

### Clinical Feature Analysis

Clinical and molecular analyses were performed in cBioPortal.^5,6^ All documented clinical features, including sex, ethnicity, age at diagnosis, performance status, and comorbidities (hypertension, insulin-dependent diabetes, chronic obstructive pulmonary disease, heart failure, and breast cancer) were evaluated. Statistical analyses were performed using Wilcoxon and chi-squared tests, and *q*-values were derived from Benjamini-Hochberg correction.

### Genomic and Transcriptomic Analyses

Sample-level enrichments were compared on the basis of alteration frequencies per gene and resultant log ratios of alterations across groups. One-sided Fisher Exact test was used for analysis of mutation frequencies between groups and q-values were derived via Benjamini-Hochberg correction. Transcriptional data were evaluated based on log ratios of mean expression levels per gene, with statistical analysis performed via student’s *t*-test. CIBERSORT^7^ was used to evaluate the presence of tumor-associated macrophages (TAMs) in male versus female samples; mean percentages of TAMs were compared using unpaired *t*-tests.

### EGFRvIII Expression

Following the identification of EGFRvIII cases (Supplementary Material), samples with and without the variant were compared on the metrics of overall survival (OS), recorded clinical features, genomic alterations, and transcriptional profiles. Transcriptomic data were analyzed utilizing the omics data processing software Ingenuity Pathway Analysis (IPA), which produced a table of drugs and experimental molecules targeting significantly altered genes.

### Survival Analysis

A Logrank test was used to compare survival across subgroups stratified by clinical or molecular features. Cases associated with both a CNS and a non-CNS diagnostic code were excluded for survival analyses and for determining median age at diagnosis, given that non-CNS tumor diagnoses always occurred prior to CNS diagnoses and OS data for those cases therefore reflected survival from earlier, non-CNS primaries. Cases were filtered to only include those with a listed vital status of “deceased” to avoid confounding effects of recently diagnosed, living patients.

For analysis of long-term survivors (LTS) versus short-term survivors (STS), patients with a non-CNS diagnostic code in addition to their CNS diagnostic code were first excluded to avoid confounding of OS data. One case lacking survival data was additionally excluded. STS cases included those with OS < 12 months and a vital status of “deceased” with cause of death “due to cancer” or “probably due to cancer” (*n* = 13). The LTS group included patients surviving past 24 months, irrespective of vital status (*n* = 69). Clinical, genomic, and transcriptomic data for LTS versus STS patients were analyzed as described above.

## Results

### Patient Characteristics

The final patient cohort consisted of 94 females and 112 males, of which 92.2% were White, 3.9% Black, 2.4% other, and 1.5% Asian or Southeast Asian. Of 9 patients with tumors reclassified as glioblastoma under 2021 WHO CNS criteria, prior diagnoses included anaplastic astrocytoma (*n* = 3), astrocytoma NOS (*n* = 2), and malignant glioma (*n* = 4). Although not significant, patients with molecularly diagnosed glioblastoma exhibited shorter median OS compared to those with histologically diagnosed glioblastoma (12.89 vs. 17.88 months). Median age at diagnosis was 58.83 years, and median OS was 18.28 months. The entire glioblastoma cohort was divided into age quartiles and compared across groups; Hypertension was the only clinical feature significantly more frequent in older patients. Sex comparison revealed no significant differences in clinical features between male and female glioblastoma patients, though males did exhibit shorter median OS (16.04 vs. 18.71 months).

### Tumor Suppressor Genes are Frequently Mutated in Germline Samples

Germline DNA samples were available for 164 patients and were analyzed for mutations in established tumor suppressor genes. The most commonly mutated tumor suppressor genes, with corresponding frequencies, were as follows: *FANCD2* (89.10%), *PRSS1* (84.60%), *FANCA* (21.80%), *ATM* (21.20%), *PMS2* (18.60%), *POLE* (18.60%), *APC* (17.90%), *BRCA2* (16.00%), *MSH3* (16.00%), *TP53* (15.40%), *BRCA1* (15.40%), *MUTYH* (15.40%), *AXIN2* (14.70%), *MSH2* (14.70%), *PTCH1* (14.10%), *MLH1* (12.80%), *MEN1* (11.50%), and *TP53BP1* (10.90%).

### Glioblastoma Cases Exhibit Frequent Mutations in a Set of 28 Genes

**Figure 2.**
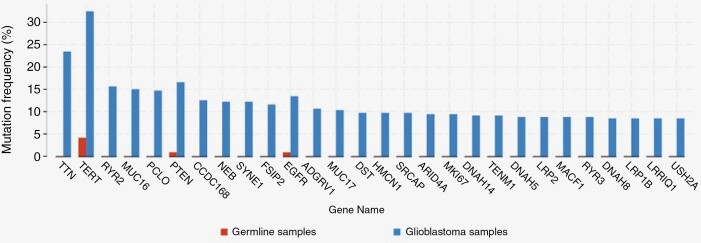
Significant mutations in glioblastoma cohort. Graph depicting genes mutated at a significantly higher frequency in glioblastoma samples in comparison to their germline counterparts, ordered by statistical significance.

In total, 348 samples from 206 patients qualified as glioblastoma via histologic or molecular characteristics. Twenty-eight genes were mutated at significantly higher rates in tumor samples compared to germline samples (Figure 2). There were no significant mutational differences between molecularly and histologically diagnosed cohorts.

### SYNE1, MUC17, and TENM1 Mutations are Associated With Longer Survival

Among the 28 significant genes, we identified 3 for which alterations were significantly associated with longer median OS: *SYNE1* (30.58 vs. 15.85 months, *P* < .01), *MUC17* (28.97 vs. 15.82 months, *P* = .01), and *TENM1* (26.57 vs. 16.10 months, *P* < .05). Further evaluation of *SYNE1* mutation samples (*n* = 38) revealed mostly missense mutations (*n* = 24; 63.16%), followed by truncating mutations (*n* = 7; 18.42%), missense mutations with gene amplification (*n* = 5; 13.16%), and splice mutations (*n* = 2; 5.26%). Samples harboring *MUC17* mutations (*n* = 33) contained primarily missense mutations (*n* = 20; 60.60%), followed by missense mutations with gene amplification (*n* = 9; 27.27%), truncating mutations (*n* = 2; 6.06%), in-frame mutations with gene amplification (*n* = 1; 3.03%), and missense mutations with deep deletion (*n* = 1; 3.03%). Analysis of *TENM1* mutations (*n* = 29) revealed missense mutations (n = 21; 72.41%), followed by missense mutations with gene amplification (*n* = 3, 10.34%), truncating mutations (*n* = 3; 10.34%), truncating mutations with gene amplification (*n* = 1; 3.45%), and missense mutations with deep deletion (*n* = 1; 3.45%).

### TERT Mutations are Associated With Older Age and Shorter Survival

Among the 28 significant genes, only mutations in *TERT* were associated with decreased OS (15.85 vs. 22.16 months, *P* = .01). *TERT* mutations were associated with older median age at diagnosis (60.9 vs. 56.9 years, *q* = 0.0281). The composition of *TERT* mutations within the entire glioblastoma cohort (*n* = 101) included promoter mutations (*n* = 89; 88.12%), promoter mutations in the setting of *TERT* amplification (*n* = 9; 8.91%), and missense mutations (*n* = 3; 2.97%).

### Specific Mutations Correlate With Differences in Overall Mutation Burden and Microsatellite Instability

Of the 28 significant genes, *TERT* mutations were associated with lower median mutation count (101.5 in mutant samples vs. 242 in wild type; *q* < 0.05) and lower median mutational burden (3.15 in mutant samples vs. 4.53 in wild type; *q* < 0.05), while *EGFR* or *PTEN* mutations exhibited no such differences. All other 25 genes were associated with increased mutational counts and burdens compared to wild-type counterparts. Mutations in *PCLO, FSIP2, DST, MKI67, RYR3, DNAH8,* and *LRRQ1* were significantly associated with increased microsatellite instability (*q* < 0.05).

### Five Transcripts Exhibit Increased Expression in Younger Patients

No mutations occurred at a significantly higher rate in any age quartile. However, 5 mRNAs were expressed at a significantly higher rate in patients aged 23.27–50.02 years: *ECD*, *DDX50*, *HIF1AN*, *CHUK,* and *IDE* (*q* < 0.05).

### Male Glioblastoma Patients Overexpress CD68

Six genes were differentially expressed between males and females (*q* < 0.05). Only one, *CD68*, was a somatic gene associated with TAMs while the rest (*NLGN4Y*, *ZFX, KDM5C, DDX3X,* and *KDM6A*) were sex-specific. CIBERSORT analysis revealed no differences in percentage of activated TAMs in male (*n* = 26 samples) versus female tumors (*n* = 27 samples; Supplementary Figure 1).

### Neither EGFR Mutation Nor Gene Amplification is Associated With OS

Of 321 samples profiled, 59 (18.4%) harbored EGFR mutations (Table 1). *EGFR* mutations were not independently associated with OS.

**Table 1.** *EGFR* Gene Mutations and Protein Changes: *EGFR* mutations and associated protein changes identified in our cohort, ordered by frequency of protein changes (VUS: Variant of Unknown Significance)

Mutation type	Copy number alteration	Driver or VUS	Protein change	# Samples with protein change
Missense	Amplification	Driver	A289V	8
Missense	Amplification	Driver	R108K	7
Missense	Amplification	Driver	R222C	4
Fusion	—	VUS	EGFR-SEPTIN14	4
Missense	Amplification	Driver	T263P	3
Missense	Amplification	Driver	G598V	3
Fusion	—	VUS	EGFR-VSTM2A	2
Missense	Amplification	Driver	A289D	1
Missense	Amplification	Driver	A289T	1
Missense	Amplification	VUS	C620F	1
Missense	Amplification	VUS	C636F	1
Missense	Amplification	VUS	D46H	1
Missense	Amplification	VUS	E282K	1
Missense	Amplification	VUS	E317Q	1
Missense	Amplification	Driver	E709K	1
Missense	Gain	VUS	E928D	1
Missense	Gain	VUS	E931K	1
Missense	Gain	VUS	G598V	1
Missense	Gain	VUS	G63R	1
Missense	Amplification	Driver	G719D	1
Missense	Amplification	Driver	G719S	1
Missense	Gain	VUS	1981F	1
Missense	Amplification	Driver	L62R	1
Missense	Amplification	Driver	P596L	1
Missense	Amplification	VUS	R324L	1
Missense	Amplification	VUS	R427H	1
Missense	Amplification	VUS	R832C	1
Missense	Amplification	VUS	S123Y	1
Missense	Amplification	VUS	S229C	1
Missense	Gain	VUS	V292M	1
Missense	Amplification	VUS	V774M	1
Missense	Amplification	Driver	R252C	1
Missense	Amplification	VUS	Y585C	1
In-Frame Insertion	Gain	Driver	772_773insH	1
Frameshift Deletion	Amplification	VUS	E204fs	1
Fusion	—	VUS	EGFR--AC011228.1	1
Fusion	—	VUS	EGFR--AC011228.2	1
Fusion	—	VUS	EGFR--AC074351.1	1
Fusion	—	VUS	EGFR--COG6	1
Fusion	—	VUS	EGFR--LINC00892	1
Fusion	—	VUS	EGFR--LINCO1446	1
Fusion	—	VUS	EGFR--MKLN1	1
Fusion	—	VUS	EGFR--MTERF1	1
Fusion	—	VUS	EGFR--PSPH	1
Fusion	—	VUS	EGFR--PSPHP1	1
Fusion	—	VUS	EGFR--SEPTIN14P8	1
Fusion	—	VUS	EGFR--VSTMA-OT1	1
Fusion	—	VUS	EGFR--ZNF804B	1
Fusion	—	VUS	FIP1L1--EGFR	1
Fusion	—	VUS	LANCL2--EGFR	1

Ninety-three tumor samples from 92 patients exhibited *EGFR* amplification, representing 56% of a cohort of 164 patients with *EGFR* copy number alteration data. Among patients with *EGFR* amplification, 35 (38%) simultaneously exhibited complete or partial gain of chromosome 7, indicating that most *EGFR* amplifications occurred independently of chromosomal copy number alteration. Survival analysis was performed on 116 patients profiled for *EGFR* copy number alteration with a vital status of “deceased” and OS data calculated from a confirmed CNS primary tumor. Patients with *EGFR* amplification (*n* = 63) exhibited nonsignificantly increased median OS compared to those without *EGFR* amplification (*n* = 53), (18.71 vs. 16.04 months, *P* = .67).

Of the 93 samples with *EGFR* amplifications, 37 additionally harbored mutations within the amplified *EGFR* gene, and 18 harbored amplifications of genes downstream in the EGFR RTK-Ras canonical signaling pathway. To analyze the survival effect of *EGFR* amplification in isolation from other *EGFR* alterations, samples with *EGFR* amplification but no mutations or downstream pathway amplifications (13 samples from 13 patients) were compared with those harboring none of the aforementioned features (36 samples from 31 patients). No significant difference in performance status or median OS was observed (12.52 months OS with *EGFR* amplification versus 15.55 months without, *P* = .69). To evaluate the added effect of *EGFR* pathway downstream amplification on OS, samples with both *EGFR* amplifications and downstream amplifications (11 samples from 11 patients) were compared with samples harboring *EGFR* amplification but lacking downstream amplification (34 samples from 34 patients). There were no significant differences in performance status or median OS between groups (23.21 months with downstream amplification vs. 18.71 months without, *P* = .61). Similarly, transcriptomic analysis comparing cases with both *EGFR* amplification and downstream pathway amplification to those with *EGFR* amplification but no downstream amplifications revealed no significant differences in EGFR RTK-Ras pathway mRNA expression.

### Higher EGFR mRNA Expression Z-Score Correlates With Decreased OS

**Figure 3.**
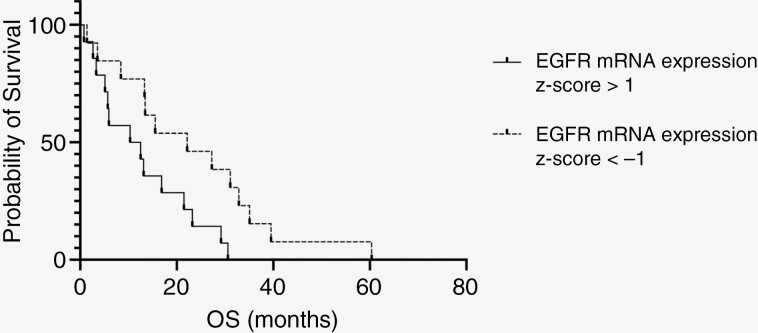
Relationship between EGFR mRNA expression and overall survival. Kaplan–Meier curve depicting survival differences between patients with increased versus decreased EGFR expression (mRNA expression z-score > 1 vs. < 1).

Utilizing *EGFR* mRNA expression z-score values, patients with EGFR expression z-scores > 1 (*n* = 14) were compared with those with z-scores < −1 (*n* = 13). Patients with EGFR mRNA expression z-scores > 1 exhibited significantly shorter OS (11.42 vs. 22.16 months, *P* = .02, Figure 3).

### EGFRvIII Expression is Associated With a Distinct Transcriptomic Profile

**Figure 4.**
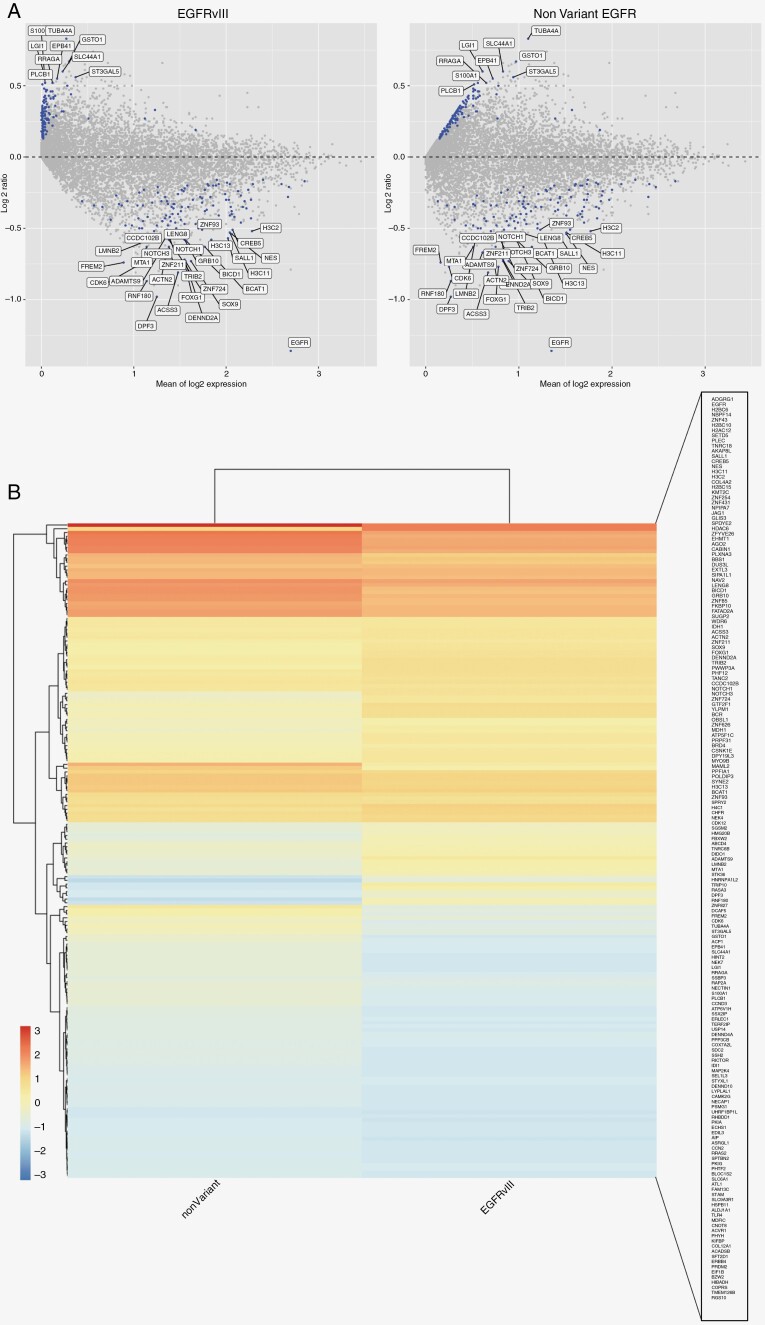
Transcriptional alterations associated with EGFRvIII expression. (A) MA plot depicting the distribution of intensity ratio over average intensity. Highlighted points signify *q* < 0.05. (B) Heatmap comparing gene expression across EGFRvIII and nonvariant samples; all genes selected for heatmap exhibited *q* < 0.05.

Of the 51 patients with RNA sequencing data available for EGFRvIII analysis, 8 samples from 8 patients expressed the variant (15.69% of cases). Although a comparison of samples harboring versus lacking the variant revealed no significant differences in clinical or genomic features, transcriptomic analysis revealed 240 transcripts differentially expressed between groups (*q* ≤ 0.05; Figure 4). Of the 240 transcripts, 67 exhibited a *q*-value of ≤0.01 (Supplementary Table 1).

Processing of the 240 transcripts using IPA revealed that EGFRvIII samples exhibited significantly higher expression of transcripts involved in the following pathways (ordered by statistical significance): DNA methylation and transcriptional repression signaling, epithelial adherens junction signaling, coordinated lysosomal expression and regulation signaling, microRNA biogenesis signaling, PPARα/RXRα activation, cAMP-mediated signaling, PTEN signaling, and ribonucleotide reductase signaling. Fifty-two canonical pathways were expressed at a significantly higher level in samples lacking EGFRvIII expression (Table 2). Transcripts associated with approved or experimental drugs are shown in Supplementary Table 2.

**Table 2.** Canonical Pathway Alterations Associated with EGFRvIII Expression: Canonical Pathways With Significant Differences in Expression Between EGFRvIII and non-EGFRvIII Samples

Canonical pathway	Higher expression in	Z-score	Associated molecules
DNA methylation and transcriptional repression signaling	EGFRvIII samples	−2.236	CDK12, CDK6, GATAD2A, H4C1, MTA1, ZEB1
PPARα/RXRα activation	EGFRvIII samples	−2.236	ACVR1, AIP, GUCY1B1, MAP2K4, PLCB1, RAP2A, RRAS2
CLEAR signaling pathway	EGFRvIII samples	−1.667	ATP6V1H, CREB5, EGFR, PPP3CB, RAP2A, RRAGA, RRAS2, TLR4, TSC2
Ribonucleotide reductase signaling pathway	EGFRvIII samples	−1.342	CDK6, CREB5, EGFR, MAP2K4, SMARCC1
Epithelial adherens junction signaling	EGFRvIII samples	−1.134	ACVR1, EGFR, NECTIN1, NOTCH1, NOTCH3, RAP2A, RRAS2
cAMP-mediated signaling	EGFRvIII samples	−0.816	CAMK2G, CREB5, GUCY1B1, PKIA, PKIG, PPP3CB, RGS10
PTEN signaling	EGFRvIII samples	−0.447	EGFR, FOXG1, INPPL1, RAP2A, RRAS2
MicroRNA biogenesis signaling pathway	EGFRvIII samples	−0.378	AGO2, DDX17, DICER1, EGFR, KHSRP, RAP2A, RRAS2
PDGF signaling	non-EGFRvIII samples	0.447	ACP1, INPPL1, MAP2K4, RAP2A, RRAS2
Superpathway of inositol phosphate compounds	non-EGFRvIII samples	0.447	ACP1, INPPL1, PLCB1, PPFIA1, PPP3CB, SSH2, STYXL1
Dopamine-DARPP32 feedback in cAMP signaling	non-EGFRvIII samples	0.447	CREB5, CSNK1E, GUCY1B1, PLCB1, PPP3CB
Regulation Of The Epithelial-mesenchymal transition by growth factors pathway	non-EGFRvIII samples	0.447	EGFR, MAP2K4, RAP2A, RRAS2, ZEB1
Pulmonary fibrosis idiopathic signaling pathway	non-EGFRvIII samples	0.577	ACVR1, CCN2, COL12A1, COL4A2, EGFR, FOXG1, JAG1, MAP2K4, NOTCH1, NOTCH3, RAP2A, RRAS2
Estrogen receptor signaling	non-EGFRvIII samples	0.707	ATP5F1C, CREB5, EGFR, FOXG1, GUCY1B1, NOTCH1, PLCB1, RAP2A, RRAS2
Autophagy	non-EGFRvIII samples	0.816	ATG5, CREB5, MAP2K4, PPP3CB, TLR4, TSC2
Glioma signaling	non-EGFRvIII samples	1	CAMK2G, CCND3, CDK6, EGFR, HDAC6, IDH1, RAP2A, RRAS2
Valine degradation I	non-EGFRvIII samples	1	ACADSB, BCAT1, ECHS1, HIBADH
UVC-induced MAPK signaling	non-EGFRvIII samples	1	EGFR, MAP2K4, RAP2A, RRAS2
UVA-induced MAPK signaling	non-EGFRvIII samples	1	EGFR, MAP2K4, PLCB1, RAP2A, RRAS2
D-myo-inositol-5-phosphate metabolism	non-EGFRvIII samples	1	ACP1, INPPL1, PLCB1, PPFIA1, PPP3CB, SSH2, STYXL1
Endocannabinoid developing neuron pathway	non-EGFRvIII samples	1	CREB5, GUCY1B1, MAP2K4, RAP2A, RRAS2
P2Y purigenic receptor signaling pathway	non-EGFRvIII samples	1	CREB5, GUCY1B1, PLCB1, RAP2A, RRAS2
Paxillin signaling	non-EGFRvIII samples	1	ACTN2, MAP2K4, RAP2A, RRAS2
NGF signaling	non-EGFRvIII samples	1	CREB5, MAP2K4, RAP2A, RRAS2
STAT3 pathway	non-EGFRvIII samples	1	EGFR, MAP2K4, RAP2A, RRAS2
GNRH signaling	non-EGFRvIII samples	1.134	CAMK2G, CREB5, EGFR, GUCY1B1, MAP2K4, PLCB1, RAP2A, RRAS2
Wound healing signaling pathway	non-EGFRvIII samples	1.134	ACVR1, COL12A1, COL4A2, EGFR, MAP2K4, RAP2A, RRAS2
Agrin interactions at neuromuscular junction	non-EGFRvIII samples	1.342	EGFR, ERBB4, MAP2K4, RAP2A, RRAS2
Neurotrophin/TRK signaling	non-EGFRvIII samples	1.342	CREB5, MAP2K4, RAP2A, RRAS2, SPRY2
Glioblastoma multiforme signaling	non-EGFRvIII samples	1.342	CCND3, CDK6, EGFR, PLCB1, RAP2A, RRAS2, TSC2
ERBB signaling	non-EGFRvIII samples	1.342	EGFR, ERBB4, MAP2K4, RAP2A, RRAS2
Insulin receptor signaling	non-EGFRvIII samples	1.342	GRB10, INPPL1, RAP2A, RRAS2, TRIP10, TSC2
Neuregulin signaling	non-EGFRvIII samples	1.342	CDK5R1, EGFR, ERBB4, RAP2A, RRAS2
Cholecystokinin/gastrin-mediated signaling	non-EGFRvIII samples	1.342	EGFR, MAP2K4, PLCB1, RAP2A, RRAS2
Factors promoting cardiogenesis in vertebrates	non-EGFRvIII samples	1.342	ACVR1, CAMK2G, CREB5, MAP2K4, PLCB1
Calcium signaling	non-EGFRvIII samples	1.342	CABIN1, CAMK2G, CREB5, HDAC6, PPP3CB, RAP2A
Thrombin signaling	non-EGFRvIII samples	1.342	CAMK2G, EGFR, GUCY1B1, PLCB1, RAP2A, RRAS2
Opioid signaling pathway	non-EGFRvIII samples	1.414	CAMK2G, CREB5, GUCY1B1, MAP2K4, PLCB1, PPP3CB, RAP2A, RGS10, RRAS2
Oxytocin signaling pathway	non-EGFRvIII samples	1.414	CREB5, EGFR, GUCY1B1, MAP2K4, PLCB1, PPP3CB, RAP2A, RRAS2
Synaptic long-term potentiation	non-EGFRvIII samples	1.633	CAMK2G, CREB5, PLCB1, PPP3CB, RAP2A, RRAS2
HIF1α signaling	non-EGFRvIII samples	1.633	CAMK2G, ELOC, MAP2K4, PPP3CB, RAP2A, RRAS2
Role of osteoclasts in rheumatoid arthritis signaling pathway	non-EGFRvIII samples	1.667	COL12A1, COL4A2, CREB5, FOXG1, MAP2K4, PPP3CB, RAP2A, RRAS2, TLR4
Chemokine signaling	non-EGFRvIII samples	2	CAMK2G, PLCB1, RAP2A, RRAS2
LPS-stimulated MAPK signaling	non-EGFRvIII samples	2	MAP2K4, RAP2A, RRAS2, TLR4
Apelin endothelial signaling pathway	non-EGFRvIII samples	2	GUCY1B1, MAP2K4, PLCB1, RAP2A, RRAS2
TGF-β signaling	non-EGFRvIII samples	2	ACVR1, MAP2K4, RAP2A, RRAS2
Oxidative phosphorylation	non-EGFRvIII samples	2	ATP5F1C, ATPAF1, COX7A2L, UQCRH
HMGB1 signaling	non-EGFRvIII samples	2	HAT1, MAP2K4, RAP2A, RRAS2, TLR4
CXCR4 signaling	non-EGFRvIII samples	2	GUCY1B1, MAP2K4, PLCB1, RAP2A, RRAS2
Role of MAPK signaling in promoting the pathogenesis of influenza	non-EGFRvIII samples	2	ATP6V1H, MAP2K4, RAP2A, RRAS2
Fc epsilon RI signaling	non-EGFRvIII samples	2	INPPL1, MAP2K4, RAP2A, RRAS2
fMLP signaling in neutrophils	non-EGFRvIII samples	2	PLCB1, PPP3CB, RAP2A, RRAS2
Hepatic fibrosis signaling pathway	non-EGFRvIII samples	2.121	ACVR1, BRD4, CCN2, CREB5, CSNK1E, MAP2K4, RAP2A, RRAS2, TLR4
14-3-3-mediated signaling	non-EGFRvIII samples	2.236	MAP2K4, PLCB1, RAP2A, RRAS2, TSC2, TUBA4A
Ferroptosis signaling pathway	non-EGFRvIII samples	2.236	H2BC10, H2BC15, H2BC5, RAP2A, RRAS2
Adipogenesis pathway	non-EGFRvIII samples	2.236	ACVR1, ATG5, HAT1, HDAC6, SOX9
Cardiac hypertrophy signaling	non-EGFRvIII samples	2.236	GUCY1B1, MAP2K4, PLCB1, PPP3CB, RAP2A, RRAS2
Senescence pathway	non-EGFRvIII samples	2.449	ACVR1, CCND3, CDK6, MAP2K4, PPP3CB, RAP2A, RRAS2
Role of NFAT in cardiac hypertrophy	non-EGFRvIII samples	2.646	CABIN1, CAMK2G, GUCY1B1, HDAC6, MAP2K4, PLCB1, PPP3CB, RAP2A, RRAS2
Cardiac Hypertrophy Signaling (enhanced)	non-EGFRvIII samples	3	ACVR1, CAMK2G, GUCY1B1, HDAC6, MAP2K4, PLCB1, PPP3CB, RAP2A, RRAS2, TSC2

### EGFRvIII Expression Does Not Significantly Influence OS

Within the subset of glioblastoma patients with RNA sequencing data available, 40 met criteria for survival analysis based on vital status and OS data, including 7 who expressed the EGFRvIII variant and 33 who did not. Patients expressing the variant exhibited a modest but nonsignificant increase in median survival (21.50 vs. 15.85 months; *P* = .93).

### LTS and STS Cohorts Demonstrate Transcriptomic Differences

To identify clinical and molecular differences between LTS and STS patients, 13 patients exhibiting STS (<12-month survival) were compared with 69 patients with LTS (>24-month survival). LTS patients were significantly younger (53.2 vs. 66.5 years; *q* < 0.001) and less likely to have documentation that medication was not part of the treatment plan (2.67% of LTS patients vs. 30.77% of STS patients; *q* < 0.01). There were no significant genomic alterations between LTS and STS patients. Transcriptomic analysis revealed 14 transcripts (*DNAJB5*, *PHTF2, TIPRL, CDC23, PGRMC2, CDKN2A, EXOSC9, MIS18BP1, RFC4, CNOT6, IQGAP2, AP3M1, ZNF521*, and *EPC1*) expressed at a significantly higher level in the LTS cohort, and two transcripts (*TMBIM1* and *CLSTN1*) expressed significantly more frequently in STS patients (*q* ≤ 0.05).

## Discussion

The present study represents the first analysis of 206 glioblastoma cases within the ORIEN brain cancer database, reclassified under updated 2021 WHO diagnostic criteria, and provides numerous novel insights into the ways genomic and transcriptomic features of glioblastoma influence clinical outcomes.

Analysis of the ORIEN glioblastoma cohort identified 28 genes mutated significantly more frequently in tumor samples; some included genes with known roles in glioblastoma including *TERT*, *PTEN,* and *EGFR*.^3^*TERT* mutations were mostly promoter mutations and were significantly associated with older age and decreased survival, corroborating findings from prior reports.^3^ Despite *PTEN* mutations also being implicated in reduced OS, our study did not identify an association between *PTEN* mutation and shorter survival.^8^

*MUC17* (mucin 17) mutations were associated with increased median OS, which represents, to the best of our knowledge, the first report of this association. *MUC17* encodes a membrane-bound protein involved in cell structure, and its protein family may play a role in extravasation of metastatic cancer cells.^9^ Contrary to our findings, a recent multi-database glioblastoma analysis found that *MUC17* mutations were associated with poorer prognosis.^10^ In breast cancer, *MUC17* knockdown improved chemosensitivity in vitro, while in lung cancer, decreased *MUC17* expression was observed in tyrosine kinase inhibitor (TKI) resistance.^11,12^ Given that EGFR TKIs have been trialed in glioblastoma with little success, the possibility that *MUC17* activity relates to TKI resistance may warrant further exploration.

*SYNE1* (synaptic nuclear envelope protein 1) mutations were also associated with prolonged survival. *SYNE1* is involved in cell cycle progression and is implicated in numerous malignancies including lung, ovarian, and colon cancers.^13^ One analysis of The Cancer Genome Atlas glioblastoma samples reported that *SYNE1* mutation was associated with higher expression of the oncogene *RAF1* and decreased expression of tumor suppressors *MTUS1, ZFHX3,* and *SPINT2*.^14^ The study additionally reported that *SYNE1* mutations were associated with mutations in mismatch repair genes *MSH6* and *MLH1*, the dysfunction of which contributes to a mutator phenotype. As in our study, another analysis of The Cancer Genome Atlas glioblastoma cases revealed an association between increased *SYNE1* expression and longer survival.^15^

Mutations in *TENM1* (tenurin transmembrane protein 1) were also associated with prolonged survival. Tenurins represent a subfamily of proteins within the tenascin family involved in CNS development, neurite outgrowth, transcriptional regulation, and interactions between cells and extracellular matrices.^16^*TENM1* mutations are associated with congenital anosmia, and previous studies have implicated *TENM1(*also known as *ODZ1)* in glioblastoma.^16–18^ One study reported that *ODZ1* enabled glioblastoma invasion via Myc-dependent upregulation of RhoA, and *ODZ1* overexpression in glioblastoma cells in xenografted mice reduced survival.^17^ Knockdown of *ODZ1* decreased glioblastoma cell invasiveness, and analysis of human glioblastoma samples revealed that *ODZ1* expression was inversely correlated with survival. Another study investigated the role of glioma-associated macrophages in promoting the invasive phenotype of glioblastoma and found that monocytic cells releasing IL-6 induced *ODZ1* expression and contributed to invasiveness via a Stat3-mediated mechanism; Blockade of this signaling pathway decreased *ODZ1* expression and glioblastoma invasiveness.^18^ These findings suggest that further investigation of *TENM1* signaling would be valuable to determine the prognostic implications of alterations in this gene in glioblastoma.

Genomic analysis between sexes revealed that male glioblastoma patients exhibited increased *CD68* mRNA expression. CD68 is a marker used to identify TAMs in cancer tissue samples, and the presence of CD68-positive cells is correlated with an immunosuppressive tumor microenvironment and worse prognosis.^19^ CIBERSORT analysis revealed no significant differences in percentages of TAMs in male versus female tumors, suggesting that differences in *CD68* expression may reflect glioma rather than immune cell *CD68* expression. Human U87 glioblastoma spheroids can express CD68 even when cultured without macrophages, and increased CD68 expression is correlated with higher tumor grade.^20,21^ Although our data did not show a significant survival difference between males and females, males exhibited slightly decreased median OS. This is consistent with previous reports demonstrating increased OS for female glioblastoma patients.^22^ One study investigating sex differences in glioblastoma linked treatment response with transcriptomic data; Female patients exhibited better overall response to standard therapies and their responses were influenced by integrin signaling pathway activity, whereas male response to therapy was influenced by cell cycle regulator expression.^23^ Sex-specific differences in gene expression may thus prove useful for prognostication in glioblastoma treatment.

This study also investigated the influence of *EGFR* alterations on glioblastoma survival. *EGFR* mutations, amplifications, and expression of the EGFRvIII variant occur frequently in glioblastoma; previous studies have reported varying results regarding the prognostic value of *EGFR* alterations.^24^ Although *EGFR* has received much attention as a potential therapeutic target, little success has been achieved via targeting *EGFR* signaling in glioblastoma. Despite *EGFR* being one of the more frequently mutated genes in our cohort, *EGFR* mutations were not associated with performance status or OS. Similarly, *EGFR* amplification was not associated with survival differences, nor was expression of the EGFRvIII variant. However, comparison of patients with higher versus lower *EGFR* mRNA expression z-scores revealed significantly shorter overall survival in those with high expression, which aligns with existing literature on *EGFR* expression in glioblastoma.^25^ Clinical evaluation of *EGFR* mRNA expression levels may potentially play a useful role in glioblastoma prognostication.

EGFRvIII expression was associated with significantly different expression of 240 transcripts compared to non-EGFRvIII samples. EGFRvIII samples were more likely to exhibit higher expression of genes involved in DNA methylation, transcriptional repression signaling, and several additional pathways. Non-EGFRvIII samples exhibited increased expression of *S100A1*, a marker of mesenchymal glioblastoma, whereas EGFRvIII samples exhibited higher expression of the classic/proneural subtype marker *NOTCH1* and the proneural-associated gene *IDH1*.^26,27^ Other notable transcriptional differences in EGFRvIII-containing samples include increased *HDAC6* expression, reported to promote glioma proliferation; increased expression of the stem cell marker *SOX9*; and increased expression of tumor suppressor *TSC2*.^26,28^ Processing the full set of 240 significant transcripts using IPA revealed numerous drugs associated with the identified transcripts, creating opportunities to hone precision medicine in glioblastoma.

EGFRvIII samples exhibited increased expression of *CDK6* and *ADAMTS9*, which are both implicated in temozolomide resistance.^29^ This finding is interesting given that EGFRvIII expression may be related to temozolomide sensitivity.^30^ To our knowledge, this represents the first study demonstrating a significant association between EGFRvIII expression and upregulation of resistance molecules such as *CDK6* and *ADAMTS9*, which creates an avenue for further investigation of combined influences of EGFRvIII, *CDK6* upregulation, and *ADAMTS9* upregulation related to temozolomide responsiveness. Given that inhibitors of CDK6 have shown promise in cell line studies and CDK6 inhibition is undergoing investigation in clinical trials, further elucidation of the relationships between EGFRvIII, resistance gene expression, and therapeutic response is recommended.^31^

Analysis of long- versus short-term glioblastoma survivors revealed 14 transcripts upregulated in the LTS cohort, representing the first report of this transcriptional signature associated with prolonged glioblastoma survival. A handful of these transcripts have been studied in various cancer models with positive prognostic value. Deletion of *CDKN2A,* a cell cycle regulator and tumor suppressor, is a known prognostic marker of poor survival in glioblastoma.^32^ It therefore follows that *CDKN2A* upregulation as observed in our study was associated with longer OS. Similarly, CDC23 is a cell cycle regulator that contributes to breakdown of mitotic proteins, and in glioblastoma models, *CDC23* knockdown is associated with increased mitotic activity.^33^ Higher *CDC23* expression was observed in the LTS cohort, consistent with existing literature characterizing the role of its protein product.

Several additional LTS genes are correlated with more favorable disease progression based on studies in other cancers, including *DNAJB5* (prostate cancer^34^), *EPC1* (head and neck squamous cell carcinoma^35^), and *AP3M1* (cervical cancer^36^). Conversely, a handful of genes in the LTS transcriptomic signature have been implicated in poorer prognosis in other cancers; these include *EXOSC9* (breast cancer^37^), *CNOT6* (osteosarcoma^38^), and *RFC4* and *PGRMC2* (numerous cancers^39–42^). *MIS18BP1* may be a microsatellite instability target gene in colorectal cancer.^43^ The exact roles of these genes in glioblastoma are not yet well-elucidated.

For other genes upregulated in the LTS cohort, the literature conflicts as to whether they are more strongly associated with poor or improved prognosis in various cancers. For example, *PHTF2* expression in esophageal squamous cell carcinoma is associated with immune infiltration and prolonged survival, but in gastric cancer, *PHTF2* contributes to tumorigenesis.^44,45^*TIPRL* appears to play a tumor-suppressive role in gastric cancer, but promotes cancer progression in lung and hepatocellular cancers.^46^*ZNF521* expression in hepatocellular carcinoma suppresses tumor growth, whereas in medulloblastoma, leukemias, and gastric cancer, it appears to increase tumorigenicity.^47^ Increased *IQGAP2* expression is implicated in colon cancer, while decreased expression is thought to contribute to gastric cancer and hormone-refractory prostate cancer.^48^ Given these mixed findings in other cancer types, further investigation into the roles of the aforementioned genes in glioblastoma will be useful to better clarify their prognostic potential.

Finally, our analysis additionally identified 2 transcripts, *TMBIM1* (transmembrane BAX inhibitor motif-containing 1) and *CLSTN1* (calsyntenin 1), that were more highly expressed in the STS cohort. Upregulation of *TMBIM1* is reported to contribute to glioblastoma proliferation and attenuated apoptosis via the p38/MAPK pathway, as well as resistance to temozolomide, and knockdown of *TMBIM1* has been demonstrated to prolong survival in animal models.^49^ The other upregulated gene in our STS cohort, *CLSTN1,* belongs to the cadherin family and is a cell adhesion molecule and postsynaptic membrane protein highly expressed in lung and ovarian cancer; its role in glioblastoma is not established.^50^ Further studies will be needed to elucidate the role of *TMBIM1 and CLSTN1* upregulation in glioblastoma.

This study benefitted from the ability to compare a sizeable cohort of glioblastoma patients using detailed clinical metrics, genomic data, and transcriptomic profiling available via ORIEN. Further, this study involved a case reclassification process per updated 2021 WHO CNS guidelines, ensuring that conclusions drawn from these data are optimally relevant. Finally, for survival analyses, cases with confounding variables (eg death from other causes or short survival datapoints reflective of recently established diagnoses) were excluded to better determine the influences of particular genomic and transcriptomic alterations on patient outcomes.

One key limitation of this study was a lack of epigenetic data available for analysis. Epigenetic changes may influence the clinical course of glioblastoma, most notably in the case of O^6^-methylguanine-DNA methyltransferase promoter methylation influencing response to temozolomide treatment.^6^ Additionally, data were collected from patients receiving care at leading cancer centers and therefore may not be representative of the broader population. Patients treated at highly resourced centers benefit from access to leading-edge technologies, multidisciplinary treatment teams, clinical trials, and other factors that contribute to improved outcomes. Finally, validation studies using larger patient cohorts, as well as functional investigations of the molecular patterns identified herein, will serve as important next steps to add context to our present findings.

In conclusion, this study represents the first comprehensive clinical and molecular analysis of glioblastoma cases in the ORIEN brain cancer database. We identify an association of *MUC17*, *SYNE1*, and *TENM1* mutations with prolonged survival; increased expression of *CD68* in male glioblastoma patients; decreased OS with increased *EGFR* mRNA expression z-score, but not *EGFR* amplification or mutation; 14 transcripts upregulated in LTS patients; and 2 transcripts (*TMBIM1* and *CLSTN1*) upregulated in STS patients. Further studies of the molecular alterations identified herein may advance the prediction of clinical outcomes and design of targeted therapeutics to enhance OS in glioblastoma.

## Supplementary Material

vdae046_suppl_Supplementary_Figure_S1_Tables_S1-S2

## Notes

*Author note*: The ORIEN Consortium is composed of 18 leading U.S. cancer centers: Moffitt Cancer Center, The Ohio State University Comprehensive Cancer Center—Arthur G. James Cancer Hospital and Richard J. Solove Research Institute, UVA Cancer Center, University of Colorado Cancer Center, University of New Mexico Comprehensive Cancer Center, Morehouse School of Medicine, Rutgers Cancer Institute of New Jersey, USC Norris Comprehensive Cancer Center, John P. Murtha Cancer Center, University of Utah Huntsman Cancer Institute, Winship Cancer Institute of Emory University, Stephenson Cancer Center at the University of Oklahoma, University of Iowa Holden Comprehensive Cancer Center, Roswell Park Comprehensive Cancer Center, University of Kentucky Markey Cancer Center, Indiana University Melvin and Bren Simon Comprehensive Cancer Center, George Washington University Cancer Center, and University of Kansas Cancer Center.
