# Supplementary material for: Profiling the molecular and clinical landscape of glioblastoma utilizing the Oncology Research Information Exchange Network brain cancer database
Source: Neurooncol Adv. 2024 Mar 27;6(1):vdae046. doi: 10.1093/noajnl/vdae046 (PMC11044707; doi:10.1093/noajnl/vdae046)
Supplement: vdae046_suppl_Supplementary_Figure_S1_Tables_S1-S2 [file vdae046_suppl_supplementary_figure_s1_tables_s1-s2.docx]

**Supplementary Information**

**Study Design**

The Oncology Research Information Exchange Network (ORIEN), established in 2014, represents a consortium of 18 U.S. cancer centers. All members of the ORIEN alliance employ a standardized Total Cancer Care® (TCC) protocol. In line with the TCC protocol, participants agree to have their clinical data followed over time, to undergo germline and tumor sequencing, and to be contacted in the future by their provider if an appropriate clinical trial or other study becomes available [1]. TCC is a prospective cohort study with a subset of patients enrolled in the ORIEN Avatar program, which involves research use only (RUO) grade whole-exome tumor sequencing, RNA sequencing, germline sequencing, and collection of deep longitudinal clinical data with lifelong follow up. Nearly 400,000 participants across the country are enrolled in TCC. Aster Insights, the commercial and operational partner of ORIEN, harmonizes all abstracted clinical data elements and molecular sequencing files into a standardized, structured format to enable aggregation of de-identified data for sharing within the Network. 555 ORIEN Avatar patients diagnosed with brain cancer from ORIEN member institutions consented to the TCC and were thus included in the study.

**Sequencing Methods (RUO)**

ORIEN Avatar specimens undergo nucleic acid extraction and sequencing at HudsonAlpha (Huntsville, AL) or Fulgent Genetics (Temple City, CA). Qiagen QIASymphony DNA purification is used for frozen and OCT tissue DNA extraction, generating an average insert size of 213 bp. Qiagen RNAeasy plus mini kit is utilized for frozen and OCT tissue RNA extraction, yielding an average insert size of 216 bp. Covaris Ultrasonication FFPE DNA/RNA kit is utilized for FFPE samples to extract DNA and RNA, generating an average insert size of 165b bp. For DNA sequencing, preparation of M2GEN Whole Exome Sequencing (WES) libraries involves hybrid capture using an enhanced IDT WES kit (38.7 Mb) with additional custom designed probes for double coverage of 440 cancer genes. Library hybridization is performed at either single or 8-plex, and sequenced on an Illumina NovaSeq 6000 instrument generating 100 bp paired reads. Tumor/normal matched samples undergo WES, with normal tissue covered at 100X and tumor tissue covered at 300X (an additional 440 cancer genes are covered at double coverage, with 200X for normal and 600X for tumor). Both tumor/normal concordance and gender identity QC checks are performed. Minimum threshold for hybrid selection is >80% of bases with >100X fold coverage for tumor samples and >50X fold coverage for normal tissue. RNA sequencing is performed using the Illumina TruSeq RNA Exome with single library hybridization, cDNA synthesis, library preparation, sequencing (100 bp paired reads at Hudson Alpha, 150 bp paired reads at Fulgent) to a coverage of 100M total reads/50M paired reads.

**Molecular Classification of Glioblastoma**

To evaluate glioma cases for inclusion as glioblastoma under molecular diagnostic criteria, the following thresholds were utilized: chromosomal gains were defined as >2 copies and losses as <2 copies, and copy number of ≥5 was used to define *EGFR* amplification. Copy numbers for both *EGFR* amplification and chromosomal gains/losses were evaluated based on whole exome sequencing data yielding copy number values for tumor samples compared to corresponding germline samples. Samples were required to contain a minimum of 30% tumor to be reclassified as glioblastoma on the basis of *TERT* promoter mutation, and a minimum of 50% tumor to be reclassified based on the presence of *EGFR* amplification or combined chromosome 7 gain and chromosome 10 loss.

**Identification of EGFRvIII expression**

For glioblastoma cases with tumor RNA-seq data available, CRAM files were converted to FASTQ format and aligned with STAR v2.7.0e to a custom GRCh37 reference containing an added EGFRvIII contig. The number of uniquely mapped reads spanning splice junctions in *EGFR* at the end of exon 1 to the beginning of exon 8 in SJ.out.tab output files were evaluated.

**Data Availability**

The data used in this research were generated through private funding by Aster Insights (www.asterinsights.com) in collaboration with the Oncology Research Information Exchange Network (ORIEN, www.oriencancer.org). Requests for access to the data used in this study can be submitted at https://researchdatarequest.orienavatar.com/.

**References**

1. Dalton WS, Sullivan D, Ecsedy J, Caligiuri MA. Patient Enrichment for Precision-Based Cancer Clinical Trials: Using Prospective Cohort Surveillance as an Approach to Improve Clinical Trials. Clin Pharmacol Ther. 2018;104(1):23–6. https://doi.org/10.1002/cpt.1051PMID:29570791

**Supplementary Data**

**Supplementary Figure 1: Differences in Percentage of Activated Macrophages in Male Versus Female Tumors.** A: Difference in percentage of M1 macrophages between sexes; B: Differences in percentage of M2 macrophages between sexes; C: Difference in M1/M2 ratios between males and females.

**
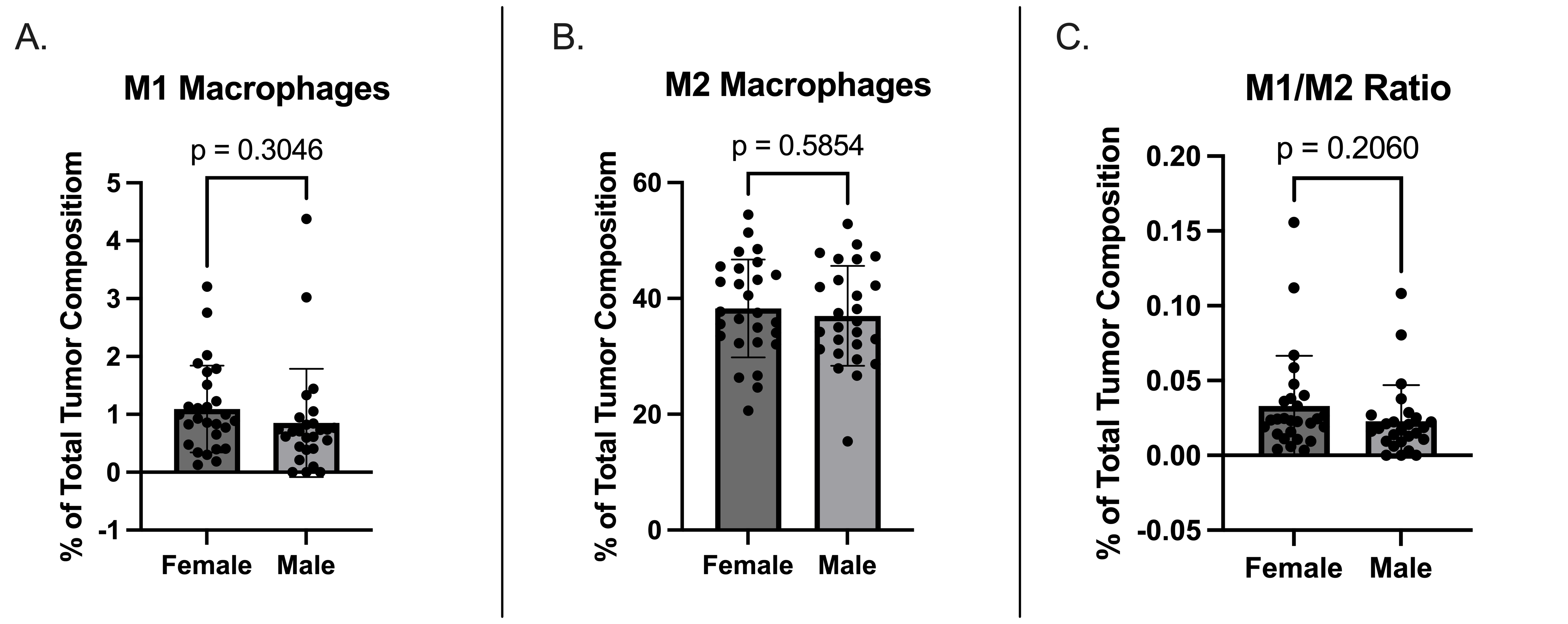
**

**Supplementary Table 1: Transcriptional Analysis of EGFRvIII Cases.** Transcripts with the most significant differences in expression between EGFRvIII and non-EGFRvIII samples (q≤ 0.01).

| **Symbol** | **Gene Name** | **Higher Expression In** | **Expr Log Ratio** | **Expr False Discovery Rate (q-value)** |
| --- | --- | --- | --- | --- |
| EGFR | epidermal growth factor receptor | EGFRvIII samples | -1.36 | 2.33E-05 |
| DENND10 | DENN domain containing 10 | non-EGFRvIII samples | 0.4 | 2.78E-05 |
| ECHS1 | enoyl-CoA hydratase, short chain 1 | non-EGFRvIII samples | 0.38 | 2.78E-05 |
| ATP6V1H | ATPase H+ transporting V1 subunit H | non-EGFRvIII samples | 0.47 | 6.62E-05 |
| CNOT8 | CCR4-NOT transcription complex subunit 8 | non-EGFRvIII samples | 0.28 | 1.77E-04 |
| EIF1B | eukaryotic translation initiation factor 1B | non-EGFRvIII samples | 0.29 | 6.18E-04 |
| PLCB1 | phospholipase C beta 1 | non-EGFRvIII samples | 0.51 | 6.18E-04 |
| STAM | signal transducing adaptor molecule | non-EGFRvIII samples | 0.33 | 6.98E-04 |
| BLOC1S2 | biogenesis of lysosomal organelles complex 1 subunit 2 | non-EGFRvIII samples | 0.36 | 7.77E-04 |
| FOXG1 | forkhead box G1 | EGFRvIII samples | -0.77 | 7.77E-04 |
| LGI1 | leucine rich glioma inactivated 1 | non-EGFRvIII samples | 0.6 | 7.77E-04 |
| CCND3 | cyclin D3 | non-EGFRvIII samples | 0.48 | 8.03E-04 |
| IDI1 | isopentenyl-diphosphate delta isomerase 1 | non-EGFRvIII samples | 0.42 | 8.03E-04 |
| MAP2K4 | mitogen-activated protein kinase kinase 4 | non-EGFRvIII samples | 0.43 | 8.03E-04 |
| ZFYVE26 | zinc finger FYVE-type containing 26 | EGFRvIII samples | -0.31 | 8.03E-04 |
| NEK4 | NIMA related kinase 4 | EGFRvIII samples | -0.33 | 8.04E-04 |
| SSX2IP | SSX family member 2 interacting protein | non-EGFRvIII samples | 0.46 | 1.01E-03 |
| SFT2D1 | SFT2 domain containing 1 | non-EGFRvIII samples | 0.32 | 1.20E-03 |
| AIP | aryl hydrocarbon receptor interacting protein | non-EGFRvIII samples | 0.34 | 1.29E-03 |
| MDFIC | MyoD family inhibitor domain containing | non-EGFRvIII samples | 0.29 | 1.35E-03 |
| ACVR1 | activin A receptor type 1 | non-EGFRvIII samples | 0.27 | 1.48E-03 |
| DENND2A | DENN domain containing 2A | EGFRvIII samples | -0.72 | 1.75E-03 |
| GOLT1B | golgi transport 1B | non-EGFRvIII samples | 0.18 | 1.75E-03 |
| PHYH | phytanoyl-CoA 2-hydroxylase | non-EGFRvIII samples | 0.3 | 2.08E-03 |
| GTDC1 | glycosyltransferase like domain containing 1 | non-EGFRvIII samples | 0.22 | 2.19E-03 |
| TNRC6B | trinucleotide repeat containing adaptor 6B | EGFRvIII samples | -0.43 | 2.35E-03 |
| LYPLAL1 | lysophospholipase like 1 | non-EGFRvIII samples | 0.37 | 2.66E-03 |
| BCAT1 | branched chain amino acid transaminase 1 | EGFRvIII samples | -0.63 | 3.14E-03 |
| SSBP3 | single stranded DNA binding protein 3 | non-EGFRvIII samples | 0.47 | 3.15E-03 |
| RRAGA | Ras related GTP binding A | non-EGFRvIII samples | 0.52 | 3.19E-03 |
| SEPTIN9 | septin 9 | EGFRvIII samples | -0.21 | 3.31E-03 |
| SLC9A3R1 | SLC9A3 regulator 1 | non-EGFRvIII samples | 0.34 | 3.31E-03 |
| NPIPA5 (includes others) | nuclear pore complex interacting protein family member A5 | EGFRvIII samples | -0.41 | 3.73E-03 |
| NOTCH1 | notch receptor 1 | EGFRvIII samples | -0.51 | 4.08E-03 |
| MDH1 | malate dehydrogenase 1 | non-EGFRvIII samples | 0.33 | 4.55E-03 |
| S100A1 | S100 calcium binding protein A1 | non-EGFRvIII samples | 0.52 | 4.55E-03 |
| RRAS2 | RAS related 2 | non-EGFRvIII samples | 0.32 | 4.73E-03 |
| ACADSB | acyl-CoA dehydrogenase short/branched chain | non-EGFRvIII samples | 0.31 | 5.10E-03 |
| ANKRD17 | ankyrin repeat domain 17 | EGFRvIII samples | -0.2 | 5.10E-03 |
| CD99 | CD99 molecule (Xg blood group) | EGFRvIII samples | -0.17 | 5.10E-03 |
| ELOC | elongin C | non-EGFRvIII samples | 0.2 | 5.10E-03 |
| ERLEC1 | endoplasmic reticulum lectin 1 | non-EGFRvIII samples | 0.36 | 5.10E-03 |
| BICD1 | BICD cargo adaptor 1 | EGFRvIII samples | -0.59 | 5.22E-03 |
| HSPB11 | heat shock protein family B (small) member 11 | non-EGFRvIII samples | 0.26 | 5.22E-03 |
| TRIB2 | tribbles pseudokinase 2 | EGFRvIII samples | -0.73 | 5.22E-03 |
| DICER1 | dicer 1, ribonuclease III | EGFRvIII samples | -0.19 | 5.80E-03 |
| NES | nestin | EGFRvIII samples | -0.54 | 5.83E-03 |
| SOX9 | SRY-box transcription factor 9 | EGFRvIII samples | -0.73 | 5.83E-03 |
| RNF170 | ring finger protein 170 | non-EGFRvIII samples | 0.23 | 6.28E-03 |
| LMNB2 | lamin B2 | EGFRvIII samples | -0.59 | 6.51E-03 |
| SIK2 | salt inducible kinase 2 | non-EGFRvIII samples | 0.17 | 7.37E-03 |
| ZNF43 | zinc finger protein 43 | EGFRvIII samples | -0.31 | 7.38E-03 |
| CCN2 | cellular communication network factor 2 | non-EGFRvIII samples | 0.34 | 8.62E-03 |
| KIFBP | kinesin family binding protein | non-EGFRvIII samples | 0.29 | 8.83E-03 |
| DUS3L | dihydrouridine synthase 3 like | EGFRvIII samples | -0.45 | 8.94E-03 |
| ATL1 | atlastin GTPase 1 | non-EGFRvIII samples | 0.32 | 9.04E-03 |
| AGO2 | argonaute RISC catalytic component 2 | EGFRvIII samples | -0.4 | 9.73E-03 |
| H3C11 | H3 clustered histone 11 | EGFRvIII samples | -0.53 | 9.73E-03 |
| INPPL1 | inositol polyphosphate phosphatase like 1 | EGFRvIII samples | -0.24 | 9.73E-03 |
| STYXL1 | serine/threonine/tyrosine interacting like 1 | non-EGFRvIII samples | 0.35 | 9.73E-03 |
| ADAMTS9 | ADAM metallopeptidase with thrombospondin type 1 motif 9 | EGFRvIII samples | -0.67 | 9.80E-03 |
| MRPL22 | mitochondrial ribosomal protein L22 | non-EGFRvIII samples | 0.23 | 9.80E-03 |
| OBSL1 | obscurin like cytoskeletal adaptor 1 | EGFRvIII samples | -0.43 | 9.80E-03 |
| ASRGL1 | asparaginase and isoaspartyl peptidase 1 | non-EGFRvIII samples | 0.35 | 9.84E-03 |
| PTTG1IP | PTTG1 interacting protein | EGFRvIII samples | -0.2 | 9.84E-03 |
| RAP2A | RAP2A, member of RAS oncogene family | non-EGFRvIII samples | 0.43 | 9.84E-03 |
| SEL1L3 | SEL1L family member 3 | non-EGFRvIII samples | 0.41 | 9.84E-03 |

**Supplementary Table 2: Drug Targets in Association with EGFRvIII-Related Gene Expression**. Genes expressed at significantly different levels between EGFRvIII and non-EGFRvIII samples that are targetable by existing drugs, revealed through Ingenuity Pathway Analysis.

| **Symbol** | **Gene Name** | **Location** | **Type** | **Expr Log Ratio** | **q-value** | **Higher Expression In** | **Drug(s)** |
| --- | --- | --- | --- | --- | --- | --- | --- |
| EGFR | epidermal growth factor receptor | Plasma Membrane | kinase | -1.36 | 2.33E-05 | EGFRvIII samples | Numerous |
| MAP2K4 | mitogen-activated protein kinase kinase 4 | Cytoplasm | kinase | 0.43 | 8.03E-04 | Non-EGFRvIII samples | cobimetinib/encorafenib, cetuximab/encorafenib, NUCC-202360, binimetinib/encorafenib, encorafenib/panitumumab, encorafenib, binimetinib/cetuximab/encorafenib, binimetinib/encorafenib/panitumumab, NUCC-226297, NUCC-201167 |
| ACVR1 | activin A receptor type 1 | Plasma Membrane | kinase | 0.27 | 1.48E-03 | Non-EGFRvIII samples | SM1-71, itacnosertib, BMP type I receptor kinase inhibitor compound A, LDN-193189, INCB000928 |
| NOTCH1 | notch receptor 1 | Plasma Membrane | transcription regulator | -0.51 | 4.08E-03 | Non-EGFRvIII samples | OMP-52M51 |
| SIK2 | salt inducible kinase 2 | Cytoplasm | kinase | 0.17 | 7.37E-03 | Non-EGFRvIII samples | HG-9-91-01 |
| CCN2 | cellular communication network factor 2 | Extracellular Space | growth factor | 0.34 | 8.62E-03 | Non-EGFRvIII samples | pamrevlumab |
| USP14 | ubiquitin specific peptidase 14 | Cytoplasm | peptidase | 0.32 | 1.02E-02 | Non-EGFRvIII samples | VLX1570 |
| CSNK1E | casein kinase 1 epsilon | Cytoplasm | kinase | -0.29 | 1.08E-02 | EGFRvIII samples | IC261, umbralisib, PF-4800567, SM1-71, WZ3105, PF-670462, SR-3029 |
| PPP3CB | protein phosphatase 3 catalytic subunit beta | Plasma Membrane | phosphatase | 0.43 | 1.16E-02 | Non-EGFRvIII samples | voclosporin, cyclosporin A, pimecrolimus, prednisone/tacrolimus, cyclosporin A/methotrexate, tacrolimus, methylprednisolone/tacrolimus |
| TLR4 | toll like receptor 4 | Plasma Membrane | transmembrane receptor | 0.27 | 1.21E-02 | Non-EGFRvIII samples | OM 174 lipid, resatorvid, GSK1795091, eritoran |
| SLC6A1 | solute carrier family 6 member 1 | Plasma Membrane | transporter | 0.35 | 1.43E-02 | Non-EGFRvIII samples | tiagabine |
| NOTCH3 | notch receptor 3 | Plasma Membrane | transcription regulator | -0.55 | 1.96E-02 | EGFRvIII samples | PF-06650808 |
| HDAC6 | histone deacetylase 6 | Nucleus | transcription regulator | -0.28 | 2.09E-02 | EGFRvIII samples | trametinib/vorinostat, KA2507, belinostat, HG146, vorinostat, ricolinostat, pyroxamide, bortezomib/vorinostat, JBI-802, tributyrin |
| ERBB4 | erb-b2 receptor tyrosine kinase 4 | Plasma Membrane | kinase | 0.32 | 2.25E-02 | Non-EGFRvIII samples | BMS-599626, allitinib, osimertinib, gefitinib/osimertinib, pelitinib, afatinib, afatinib/dasatinib, afatinib/paclitaxel, pirotinib, carbon C 14 dacomitinib, afatinib/cetuximab, mobocertinib, epertinib, erlotinib/osimertinib, neratinib/paclitaxel, FCN-411, JNJ-26483327, poziotinib, dacomitinib, neratinib, afatinib/osimertinib |
| BCR | BCR activator of RhoGEF and GTPase | Cytoplasm | kinase | -0.48 | 2.56E-02 | EGFRvIII samples | BCR targeting agent |
| COL4A2 | collagen type IV alpha 2 chain | Extracellular Space | other | -0.46 | 2.59E-02 | EGFRvIII samples | collagenase |
| TUBA4A | tubulin alpha 4a | Cytoplasm | other | 0.83 | 2.74E-02 | Non-EGFRvIII samples | Numerous |
| STK36 | serine/threonine kinase 36 | Cytoplasm | kinase | -0.5 | 3.13E-02 | EGFRvIII samples | cobimetinib/encorafenib, cetuximab/encorafenib, binimetinib/encorafenib, encorafenib/panitumumab, encorafenib, binimetinib/cetuximab/encorafenib, binimetinib/encorafenib/panitumumab |
| BRD4 | bromodomain containing 4 | Nucleus | kinase | -0.32 | 3.19E-02 | EGFRvIII samples | JAB-8263, AZD5153, PLX51107, AX15839, SYHA1801, PLX2853, NUV-868, BI 894999, AX15910, SF2523 |
| COL12A1 | collagen type XII alpha 1 chain | Extracellular Space | other | 0.31 | 3.41E-02 | Non-EGFRvIII samples | collagenase |
| ALDH1A1 | aldehyde dehydrogenase 1 family member A1 | Cytoplasm | enzyme | 0.26 | 3.97E-02 | Non-EGFRvIII samples | disulfiram, chlorpropamide |
| CDK6 | cyclin dependent kinase 6 | Nucleus | kinase | -0.77 | 3.98E-02 | EGFRvIII samples | fulvestrant/ribociclib, abemaciclib/anastrozole, trilaciclib, abemaciclib, CINK4, XZP-3287, exemestane/palbociclib, TQB3303, CS3002, dalpiciclib, lerociclib, ribociclib/tamoxifen, palbociclib, BPI-1178, TY-302, fulvestrant/palbociclib, BPI-16350, HS-10342, abemaciclib/aromatase inhibitor, abemaciclib/exemestane, FCN-437, GLR2007, FLX925, ribociclib, FN-1501, anastrozole/ribociclib, JNJ-7706621, everolimus/ribociclib, abemaciclib/fulvestrant/GnRH analog, letrozole/ribociclib, PD 0183812, abemaciclib/aromatase inhibitor/GnRH analog, abemaciclib/fulvestrant, NUV-422, TQB3616, narazaciclib, letrozole/palbociclib, anastrozole/palbociclib, abemaciclib/letrozole, alvocidib, exemestane/ribociclib |
| IDH1 | isocitrate dehydrogenase (NADP(+)) 1 | Cytoplasm | enzyme | -0.33 | 4.02E-02 | EGFRvIII samples | olutasidenib, IDH305, ivosidenib, DS-1001, HMPL-306, BAY1436032, KY100001, IDH1 inhibitor, LY3410738 |
| GUCY1B1 | guanylate cyclase 1 soluble subunit beta 1 | Cytoplasm | enzyme | 0.18 | 4.25E-02 | Non-EGFRvIII samples | nitroprusside, isosorbide-5-mononitrate, isosorbide dinitrate |
| PLEC | plectin | Cytoplasm | other | -0.28 | 4.33E-02 | EGFRvIII samples | ZB131 |
| CDK12 | cyclin dependent kinase 12 | Nucleus | kinase | -0.34 | 4.53E-02 | EGFRvIII samples | THZ531, SR4835 |
| PRMT1 | protein arginine methyltransferase 1 | Nucleus | enzyme | -0.2 | 4.57E-02 | EGFRvIII samples | GSK3368715, PRMT1 inhibitor |
| DDR1 | discoidin domain receptor tyrosine kinase 1 | Plasma Membrane | kinase | -0.23 | 4.58E-02 | EGFRvIII samples | imatinib/sirolimus, imatinib/sorafenib, nilotinib/rituximab, corticosteroid/nilotinib, imatinib/nilotinib, imatinib/inotuzumab ozogamicin, SM1-71, imatinib/sunitinib, WZ-4-145, dexamethasone/nilotinib/vincristine, dacomitinib, blinatumomab/imatinib, DDR1-IN-1, bosutinib/imatinib, dasatinib/nilotinib, ICP-033, decitabine/imatinib, nilotinib, imatinib, imatinib/rituximab, pralsetinib |
